# MYH7-related myopathies: clinical, histopathological and imaging findings in a cohort of Italian patients

**DOI:** 10.1186/s13023-016-0476-1

**Published:** 2016-07-07

**Authors:** C. Fiorillo, G. Astrea, M. Savarese, D. Cassandrini, G. Brisca, F. Trucco, M. Pedemonte, R. Trovato, L. Ruggiero, L. Vercelli, A. D’Amico, G. Tasca, M. Pane, M. Fanin, L. Bello, P. Broda, O. Musumeci, C. Rodolico, S. Messina, G. L. Vita, M. Sframeli, S. Gibertini, L. Morandi, M. Mora, L. Maggi, A. Petrucci, R. Massa, M. Grandis, A. Toscano, E. Pegoraro, E. Mercuri, E. Bertini, T. Mongini, L. Santoro, V. Nigro, C. Minetti, F. M. Santorelli, C. Bruno, on behalf of the Italian Network on Congenital Myopathies

**Affiliations:** IRCCS Stella Maris, Molecular Medicine and Neuromuscular Disorders, Via dei Giacinti 2, 56128 Calambrone, Pisa Italy; Telethon Institute of Genetics and Medicine, Naples, Italy; Unit of Pediatric Neurology and Muscular Disorders, Istituto G.Gaslini, Genoa, Italy; Department of Neurosciences and Reproductive and Odontostomatologic Sciences, University Federico II, Naples, Italy; Department of Neurosciences “Rita Levi Montalcini”, University of Turin, Turin, Italy; Unit of Neuromuscular and Neurodegenerative Disorders, Department of Neurosciences, IRCCS Bambino Gesù Children’s Hospital, Rome, Italy; Don Carlo Gnocchi ONLUS Foundation, Rome, Italy; Department of Paediatric Neurology, Catholic University, Rome, Italy; Department of Neurosciences, University of Padua, Padua, Italy; Department of Clinical and Experimental Medicine and Nemo Sud Clinical Centre, University of Messina, Messina, Italy; Neuromuscular Diseases and Neuroimmunology Unit, IRCCS Foundation C Besta Neurological Institute, Milan, Italy; Center for Neuromuscular and Neurological Rare Diseases, S. Camillo-Forlanini Hospital, Rome, Italy; Department of Systems Medicine (Neurology), University of Tor Vergata, Rome, Italy; Department of Neuroscience, Rehabilitation, Ophthalmology, Genetics, Maternad and Child Health, University of Genova, University of Genoa, Genoa, Italy; Department of Neuroscience, Center of Myology and Neurodegenerative Disorders, Istituto Giannina Gaslini, Genoa, Italy

**Keywords:** Myosin heavy chain, Distal myopathy, Muscle MRI, Muscle biopsy, Whole exome sequencing

## Abstract

**Background:**

Myosin heavy chain 7 (*MYH7*)-related myopathies are emerging as an important group of muscle diseases of childhood and adulthood, with variable clinical and histopathological expression depending on the type and location of the mutation. Mutations in the head and neck domains are a well-established cause of hypertrophic cardiomyopathy whereas mutation in the distal regions have been associated with a range of skeletal myopathies with or without cardiac involvement, including Laing distal myopathy and Myosin storage myopathy. Recently the spectrum of clinical phenotypes associated with mutations in *MYH7* has increased, blurring this scheme and adding further phenotypes to the list. A broader disease spectrum could lead to misdiagnosis of different congenital myopathies, neurogenic atrophy and other neuromuscular conditions.

**Results:**

As a result of a multicenter Italian study we collected clinical, histopathological and imaging data from a population of 21 cases from 15 families, carrying reported or novel mutations in *MYH7*. Patients displayed a variable phenotype including atypical pictures, as dropped head and bent spine, which cannot be classified in previously described groups. Half of the patients showed congenital or early infantile weakness with predominant distal weakness. Conversely, patients with later onset present prevalent proximal weakness. Seven patients were also affected by cardiomyopathy mostly in the form of non-compacted left ventricle. Muscle biopsy was consistent with minicores myopathy in numerous cases. Muscle MRI was meaningful in delineating a shared pattern of selective involvement of tibialis anterior muscles, with relative sparing of quadriceps.

**Conclusion:**

This work adds to the genotype-phenotype correlation of *MYH7*-relatedmyopathies confirming the complexity of the disorder.

**Electronic supplementary material:**

The online version of this article (doi:10.1186/s13023-016-0476-1) contains supplementary material, which is available to authorized users.

## Background

The *MYH7* gene on chromosome 14 encodes slow/b-cardiac myosin heavy chain (MyHCI), a class II myosin expressed in slow, type 1 muscle fibers as well as in the heart ventricles. MyHCI is the molecular motor of muscle and forms the backbone of the sarcomere thick filaments [1].

As expected both cardiac and skeletal muscle disorders can arise from a defect of *MYH7*. MYH7- related cardiac diseases are more frequent and include familial hypertrophic/dilated cardiomyopathy (MIM 192600), and left ventricular non-compaction (LVNC) cardiomyopathy (MIM 613426) with more than 200 mutations described so far [2].

Skeletal myopathies due to *MYH7* mutations were initially classified in two main subgroups according to clinical and pathological findings: myosin storage myopathy (MSM, MIM 608358) and Laing distal myopathy (LDM, MIM 160500). Additional phenotypes have been reported over the years, including a scapulo-peroneal or limb-girdle muscle form with or without morphological features of myosin storage [3], multi-minicore disease with variable cardiac involvement [4, 5] and families with morphological features suggestive of congenital fiber type disproportion [6, 7]. These disorders are either due to *de novo* mutations or inherited in a dominant fashion although cases with autosomal recessive inheritance have seldom been reported [8, 9].

Slow/b-cardiac myosin in vivo exists as a dimer of two globular heads attached to a long, α-helical coiled-coil region. The N-terminus heads bind actin and ATP, which is required for motor activities. The C-terminus rod assembles myosin into thick filaments of the sarcomere through charge-based interactions between adjacent rods [10]. Mutations accounting for the cardiac or skeletal muscle disorders cluster in different parts of the protein: most cardiomyopathy related mutations are located in the globular head domain potentially affecting the binding sites for actin [2] whereas those linked to skeletal myopathy are usually located in the distal regions of the rod domain (also called light meromyosin domain -LMM). In particular, mutations that cause myosin storage myopathy (MSM) affect the very distal end of the tail, (exons 37–40) whereas most but not all patients reported with LDM display mutations in exons 32-36 in the mid region of *MYH7* [11]. However, there is a greater than expected overlap of phenotypes as cases with both heart and muscular manifestations are described.

Here we report clinical, morphological, and myoimaging data from 21 patients with confirmed mutations in *MYH7* to better characterize the emerging genotype–phenotype correlation in these myopathies.

## Methods

Patients were recruited from a multicenter study on congenital myopathies which gathered 13 Italian tertiary care centers for pediatric and adult neuromuscular disorders. Written informed consent was obtained from all subjects or their legal guardians when primary diagnostic procedures were performed, with explicit consent for future use for research purposes and publication of images. All patients underwent a systematic clinical characterization by at least two experts in neuromuscular disorders, including neurological, cardiac (ECG and echocardiogram), and respiratory (spirometer) assessments. CK level was recorder in all patients. Follow up ranged from 5 to 30 years. Neurophysiological studies (EMG and nerve conduction velocity) were performed in 12 patients.

In 9 cases the presence of suggestive clinical and histopathological signs directly targeted the Sanger sequencing of the coding regions of *MYH7*. Twelve cases were diagnosed as unspecific myopathies and, in absence of disease markers, underwent Motorplex analysis, a targeted molecular tool in next-generation sequencing (NGS) designed to analyze 93 genes known to be responsible of different inherited myopathies [12].

The experimental strategies, variants annotation and prioritization in Motorplex were as described elsewhere [13]. In particular, we used different prediction algorithms such as PolyPhen, SIFT e Mutation Taster to corroborate the causative effect of novel variants identified. To predict the splicing effect of splice-site variants, we consulted http://www.fruitfly.org/seq_tools/splice.html.

All the mutations identified in *MYH7* were confirmed by Sanger sequencing. Segregation analysis was performed using DNA from all available family members.

RNA from the skeletal muscle biopsies in patients 6 and 19 was extracted using TRIzol reagent according to the manufacturer’s instructions (Invitrogen, Carlsbad, CA, USA). Messenger RNA was retrotranscribed using the SuperScript III kit (Invitrogen). For the cDNA analysis, the following primers were used: 36_37F: 5'-AAGGCCATCACGGATGCC; 3'UTR_R: 5'-CCTCAAGGGCGGCAAGAA.

Having reached a confirmatory molecular diagnosis, we reviewed the muscle histology features in 16 patients adopting standard histological and histochemical stains. In four patients, we also performed immunohistochemical analyses to study the expression of slow MyHC (NCL-MHCs Novocastra Lyophilized Mouse Monoclonal Antibody).

Muscle MRI was performed in 14 patients according to conventional protocol examining axial planes of the pelvis and lower limbs with T1-weighted spin echo sequences. All muscle MRI imaging data were reviewed and scored by two experts (EM, GT) in the network.

*In silico* prediction of the effect of novel mutations falling in the LMM region was performed using the COILS program [14], a web-based tool which compares a specific amino acidic sequence to a database of already known parallel two-stranded coiled-coils. By using a similarity score, COILS is able to calculate the probability of a coiled-coil conformation. In particular, a MTIDK matrix derived from myosins, paramyosins, tropomyosins, intermediate filaments type I - V, desmosomal proteins and kinesins scanning windows of 14, 21 and 28 amino acidic residues was used to calculate the probability of coiled coil formation for the wt sequence and 4 selected missense mutations in the LMM domain.

## Results

### Patients

**Fig. 1:**
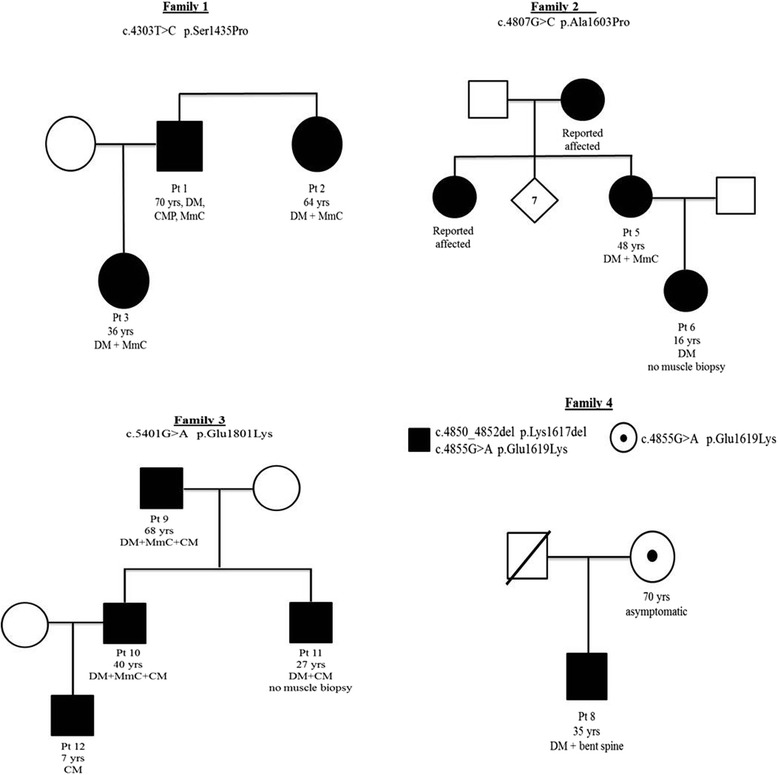
Pedigrees of the familial cases

Twenty-one patients are described. Twelve are women and nine men. Age varied between 7 and 70 years. Age at onset was between 0 and 49 years. Age at diagnosis was between 6 and 68 years. Nine cases were sporadic, nine displayed positive family history (Fig. 1) whereas in two further cases a positive family history was reported but possibly affected relatives were not available for clinical examination nor for genetic analysis. Another patient (case 8) had two mutations, one inherited from the asymptomatic mother (Family 4).

### Clinical presentation

**Fig. 2:**
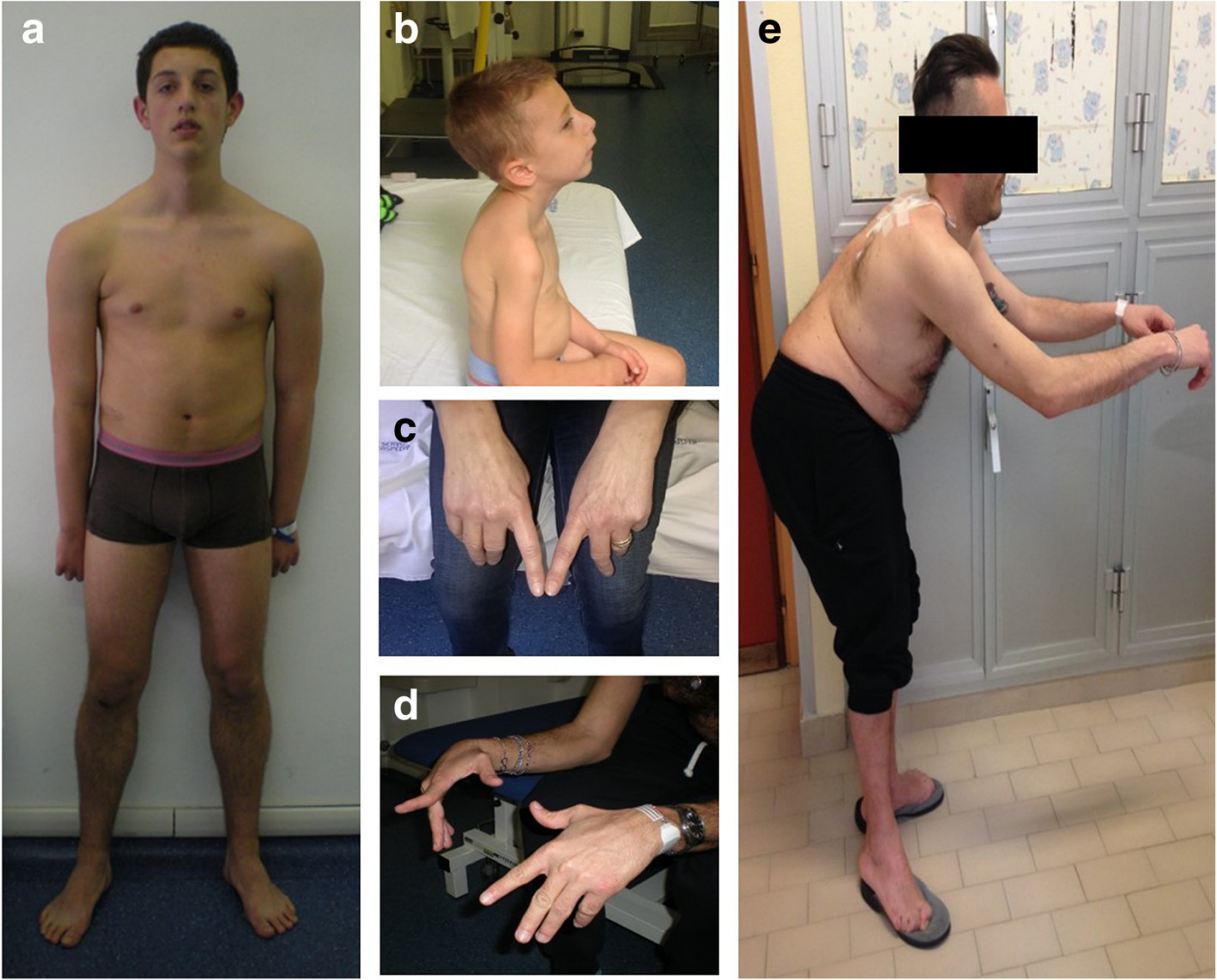
Clinical images from selected cases. **a**: case 4 at 16 years old presenting a distal phenotype with weakness and atrophy of upper limbs whereas shoulder girdle is normal. Note mild ptosis and calf hypertrophy. **b**: case 7 at 5 years of age. Strength is preserved except in axial muscle with dropped head. Mild ptosis is also present. The boy was also diagnosed with non-compacted left ventricle. **c** and **d**: two pictures of hands weakness (case 5 and 8) particularly affecting third, fourth and fifth fingers whereas second and first finger are spared. As consequence, patients have difficulties in raising the ulnar part of the hand. In (**e**) the bent appearance of the spine in patients 8. Note also the severe distal atrophy of lower limbs for which the patient had a diagnosis of motor neuropathy

Detailed clinical data of patients are summarized in Table 1. Five patients presented onset at birth or in the first year of life, four patients had onset during childhood, whereas most patients (9/21) had adult onset of the muscle weakness (>18 years), though in a single case arched palate had been reported since infancy (case 21). One patient (case 12) aged 7 years, was affected only by cardiomyopathy without muscle weakness. Heart impairment was revealed only by a positive family history (Family 3). Weakness of distal muscle was shared by all the patients, except case 7 who displayed only weakness of neck flexor when examined at age 8. Nine patients had only distal leg involvement with tibialis anterior weakness. Toe dropping was one of the first sign recalled at onset for seven patients (Table 1). In eight patients hands weakness was also present and this was the predominant feature in five cases, however this was not present at onset. Wrist extensor and fourth- fifth fingers extensor muscles (ulnar side) were particularly affected, whereas thumb abduction and second finger extension were less involved, giving a peculiar sign of pointed second finger (Fig. 2c-d). In nine cases there was also proximal muscle weakness. Axial weakness was a common finding present in 11 cases and nine patients had scoliosis, whereas two presented rigid spine. Foot deformities were noticed in nine cases, two had club foot since infancy, whereas seven patients developed pes cavum and hammertoe later with the disease progression. Facial weakness was variably present with ptosis (three cases) and elongated face (two cases). Disease can be severe (5/21) and was usually progressive over time, however most patients were still ambulant at time of diagnosis. Two patients displayed respiratory involvement and required mechanical non-invasive ventilation from age 7 and 16 years. Two more patients had mild respiratory dysfunction restrictive type with FVC reduction since late teen.

**Table 1:** Clinical, histopathological and genetic features

#		Age	Family history	Onset of symptoms	Weakness distribution	Scoliosis	Respiratory involvement	Cardiac involvement	CK U/l	EMG	Muscle biopsy	Mutation
1	M	70	yes	adult	proximal and distal lower limb	no	no	CMH	944	myopathic	cores	c.4303 T > C p.Ser1435Pro
				TA weakness								
2	F	64	yes	adult	axial, proximal and distal lower limb	yes	no	no	normal	myopathic	cores	c.4303 T > C p.Ser1435Pro
				TA weakness								
3	F	36	yes	adult	distal lower limb	no	no	no	normal	myopathic	na	c.4303 T > C p.Ser1435Pro
4	M	22	no	childhood	axial, distal upper and lower limbs	rigid spine	yes	recurrent pericarditis	711	na	minicores	c.4850_4852del p.Lys1617del
				TA weakness								
							mild restrictive from age 16					
5	F	48	yes	childhood	distal upper and lower limbs	yes	no	no	normal	na	minicores	c.4807G > C p.Ala1603Pro
				TA weakness								
6	F	16	yes	congenital	axial, distal upper and lower limbs	yes	no	no	normal	na	na	c.4807G > C p.Ala1603Pro
7	M	8	no	congenital	axial	yes	no	LVNC	normal	myopathic	minicores	c.5655 + 1G > A p.1854_1885del
8	M	35	no^a^	childhood	axial and distal	yes	no	no	720	myopathic and neurogenic	cores	c.4850_4852del p.Lys1617del c.4855G > A p.Glu1619Lys
				TA weakness								
9	M	68	yes	adult	axial proximal distal	yes	no	LVNC and PM	400-500	myopathic	FTD	c.5401G > A, p.Glu1801Lys
10	M	40	yes	adult	proximal and distal lower limb	no	no	LVNC and PM	400-500	myopathic	FTD	c.5401G > A, p.Glu1801Lys
11	M	38	yes	adult	proximal and distal lower limb	no	no	LVNC	400-500	na	na	c.5401G > A, p.Glu1801Lys
12	M	7	yes	no muscle symptom	normal	no	no	LVNC	1400	na	na	c.5401G > A, p.Glu1801Lys
13	F	59	yes	adult	proximal and distal lower limb	rigid spine	no	no	normal	myopathic	cores	c.4315G > C p.Ala1439Pro
				proximal onset								
14	F	58	no	adult	proximal upper and lower limb	na	no	no	450	myopathic and neurogenic	unspecific	c.4363G > T, p.Glu1455X
15	F	20	no	childhood	axial and distal lower limb	yes	yes	no	normal	na	unspecific	c.4475 T > C p.Leu1492Pro
							NIV needed from age 17					
16	F	39	no	adult	axial proximal distal	no	no	no	normal	myopathic	na	c.1780C > A p.Leu594Met
				hands onset								
17	M	36	no	childhood	axial and distal upper and lower limb	no	no	no	normal	na	unspecific	c.5779A > T, p.Ile1927Phe
18	F	18	no	congenital	axial and distal lower limb	yes (severe)	yes	left bunlde block	normal	myopathic	unspecific	c.5655G > A, p.Ala1885Ala
							NIV needed from age 7					
19	F	33	yes	childhood	proximal and distal upper and lower limb	yes	yes	no	normal	myopathic	minicores	c.4850_4852del, p.Lys1617del
				TA weakness			mild restrictive from age 25					
20	F	39	no	adult	distal axial and proximal	no	no	no	normal	myopathic	cores	c.5808G > C, p.X1936Tyr
21	F	40	no	adult	distal	no	no	no	500	myopathic	hyalin bodies	c.1322C > T p.Thr441Met
				TA weakness								

*FTD* fibre type disproportion, *NIV* non invasive ventilation, *LVNC* left ventricular non compaction, *PM* pace-maker, *CMH* cardiomyopathy hypertrophic. ^a^ case 8 reported negative family history albeit his mother actually carries one mutation of MYH7 but shows no symptoms of myopathy nor cardiac disease

Heart was affected in seven cases (all men), and ranged from bundle branch block to overt cardiomyopathy with features of non-compaction of left ventricle. Two patients required a pace-maker. Another case (case 5) suffered from episodes of pericarditis. Serum CK levels were normal or mildly elevated (below 1500 U/l) in all patients. EMG was usually myopathic, however three patients (cases 8, 14, and 21) presented also neurogenic features such as polyphasic and increased amplitude motor potentials and two (cases 8 and 14) had been diagnosed with a motor neuropathy for several years before genetic studies allowed the correct diagnosis.

### Atypical features

**Fig. 3:**
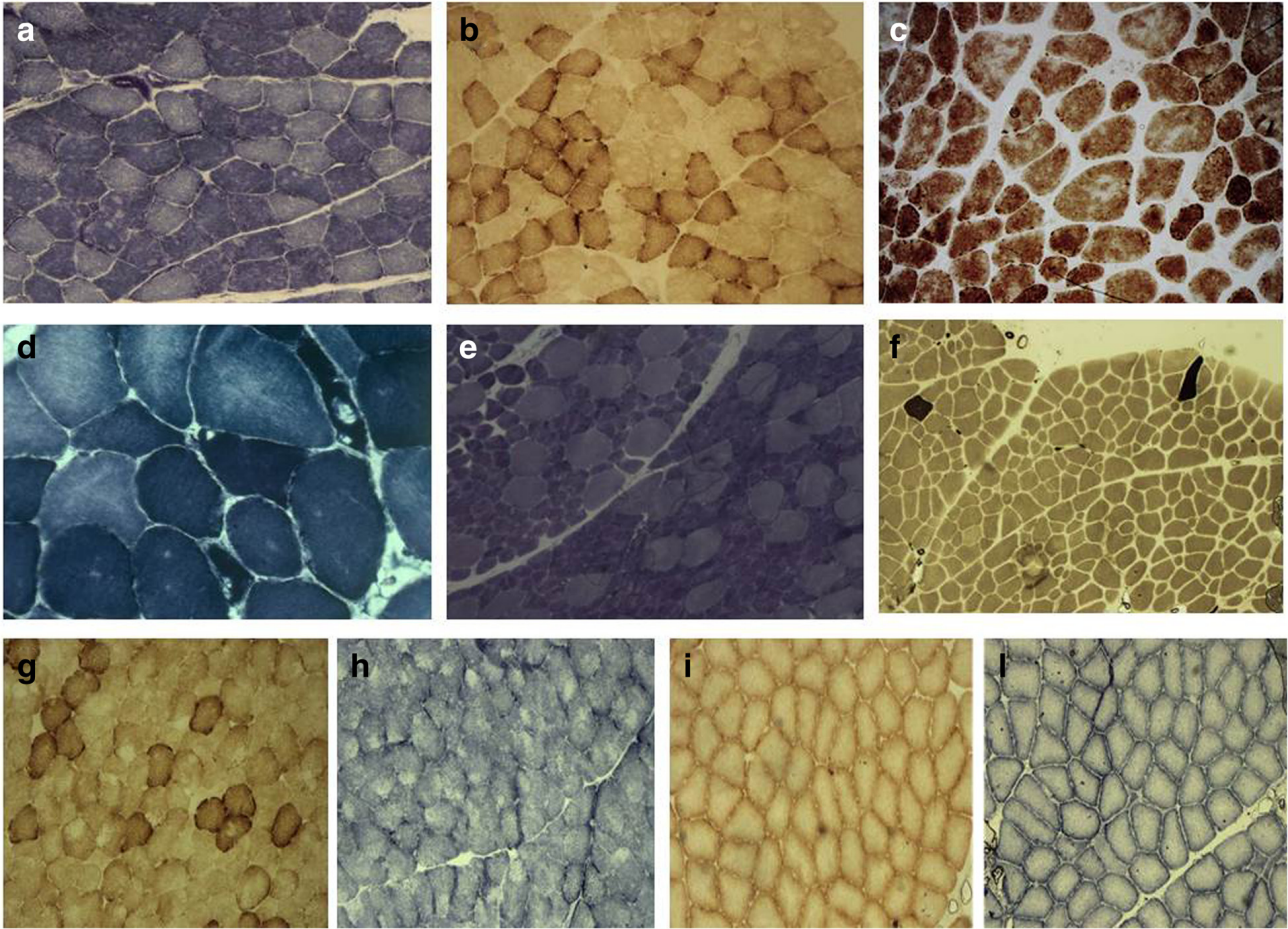
Muscle biopsy spectrum. **a** and **b**: Case 7: NADH and COX stainings, respectively showing presence of mutiple cores (magnification 20x). **c**: Case 8: COX staining of muscle sections showing severe alteration of intermyofibrillar network such as moth eaten fibres (magnification 40x). **d**: Case 21: NADH staining displaying hyaline bodies (magnification 40x). **e**: Case 10. This case has already been described in reference Ruggiero et al. NADH staining presenting fibre type disproportion with small type 1 fibres representing the majority of fibres and larger type 2 fibres (magnification 10x). **f**: Case 8. ATP 9.6 (magnification 10x) reveals pronounced type 1 predominancy or nearly uniformity. Most fibres are type 1 with only few scattered type 2 fibres. **g**-**l**: Case 4 had two muscle biopsies taken at different ages. In the first biopsy at age 7 years, COX (**g**) and NADH (**h**) staining showed clear cores in numerous fibres. The same staining (**i** and **l**) did not show cores, however the inter-myofibrillar network appeared diffusely pale and thin in the second biopsy performed ten years later

Case 4 is an 8-year-old boy who came to medical attention at the age of 4 for dropped head due to isolated weakness of neck extensors (Fig. 2b). Muscle biopsy displayed several minicores (Fig. 3 a-b). Molecular analysis of SEPN1 and LMNA was performed and mutations excluded. Follow up evaluation at age 8 revealed also hypertrophy of gastrocnemii and heart ultrasound discovered signs of left ventricular non compaction. This together with the presence of calves hypertrophy and the absence of rigid spine could have better targeted the genetic investigation.

Case 8 is a 35-year-old man with longstanding diagnosis of motor neuropathy predominantly affecting the lower limbs. This patient developed in his 20s a progressive weakness of axial muscle ending in a bent spine phenotype (Fig. 2e). Interestingly he carried two mutations in *MYH7*, one inherited from his 70-year-old mother who is clinically normal (Fig. 1 Family 4). Thus, in patient 8 we cannot totally rule out an AR pattern of inheritance.

Case 12 is a 7-year-old boy, the son of case 10 in family 3. He presented with only elevated serum CK when the father received a molecular diagnosis. Further investigations revealed signs of LVNC cardiomyopathy but neurological examination is normal. Genetic analyses confirmed that he carried the same mutation in *MYH7*.

Cases 15 and 18 have an unremarkable family history and share a similar clinical phenotype characterized by early onset congenital myopathy and marked respiratory muscles involvement requiring pulmonary support. Case 18 also has arched palate. Both patients showed normal serum CK levels and a prevalent weakness in the axial muscle with scoliosis. The patients carry different mutations localized in the same LMM domain

### Familial variability

Nine patients belong to three different families presenting an autosomal dominant pattern of transmission. Family pedigrees are in Fig. 1.

Family 1 comprises two affected siblings, one 70-year-old man (case 1) and one 64-year-old woman (case 2) and the 35-year-old daughter of case 1. All family members share a similar phenotype characterised by late onset distal myopathy mainly affecting lower limbs with subsequent involvement of proximal limbs in the two eldest patients. In no case there was respiratory involvement; however case 1 also displays hypertrophic cardiomyopathy. Muscle biopsy displayed hypotrophy of type 1 fibres and presence of cores like area.

Family 2 consists of a 48-year-old woman (case 5) and her 16-year-old daughter (case 6). An elder sister of case 5 and her 93-year-old mother were reportedly affected by a severe scoliosis and tetraparesis with loss of ambulation; they were not available for our clinical examinations. Case 5 has seven unaffected siblings and shows an early onset distal myopathy with typical bilateral hand involvement. Her daughter, case 6, has mainly axial and distal myopathy. Interestingly, face weakness and scoliosis were also prominent in this family, whereas there were no pulmonary or heart involvements. Muscle biopsy performed only in the mother showed marked uniformity of fibre type and presence of diffuse mini-cores.

Family 3 has previously been described elsewhere by same authors [7]. Briefly, this is a three-generation family with four affected men and a frequent cardiac involvement and mild-to-moderate weakness in distal upper and lower limbs which worsened with age. Two patients required permanent pacemaker. As for other relatives, an additional patient (case 12) with non compacted left ventricle was recently detected in this family. This boy had initially escaped molecular testing because he was asymptomatic at the time of our first report. A subsequent ECG recording prompted *MYH7* testing and heart ultrasound revealed the presence of non compacted left ventricle. Neurological investigation is currently normal at the age of 7 years.

In Family 4, case 8 harbors the c.4855G > A/p.Glu1619Lys on the maternal allele in compound heterozygosity with c.4850_4852del p.Lys1617del. Parents are reportedly healthy, though the father had died before our studies because of a cancer. Although the missense variant affecting residue 1619 has already been associated with familial dilated cardiomyopathy [15] and it appears to modify myosin protein structure upon studies in silico with COILS, we cannot firmly established that this variant is pathogenic and cannot conclude on the pattern of transmission in this patient.

### Histopathology

Muscle sections were available for revision in 16 cases (Additional file 1: Table S2). Cores and mini-cores were found in nine patients. Other common findings included centrally located nuclei (eight cases) and type 1 predominance (in four) or fibre type disproportion (FTD) in three patients (in two families), defined by percentage of type 1 fibre greater that 80 % and average diameter of type 1 fibre smaller than type 2 fibre of 30 %. In three further muscles biopsies a selective hypotrophy of type 1 fibres was detected. We also observed that connective tissue could be increased, especially if the biopsy is taken at later stage of the disease. Case 4 underwent two consecutives muscle biopsies: the first at 7 years old showed significant cores and minicores (Additional file 2: Figure S8), whereas the second biopsy at 15 years taken from the contralateral muscle, cores were no longer evident (Fig. 3g-i). Hyaline bodies which are typical of MSM were looked for and encountered only in case 21 (Fig. 3d), whereas case 5 presented a single rimmed vacuole in a minicore myopathy muscle biopsy. Of note both patients performed biopsy in adulthood.

Myosin staining with specific antibodies was also performed in four patients (case 4,5,7,and 8) to test for myofibrillar alteration and demonstrated a normal expression of slow myosin in myofilaments.

### Muscle MRI

**Fig. 4:**
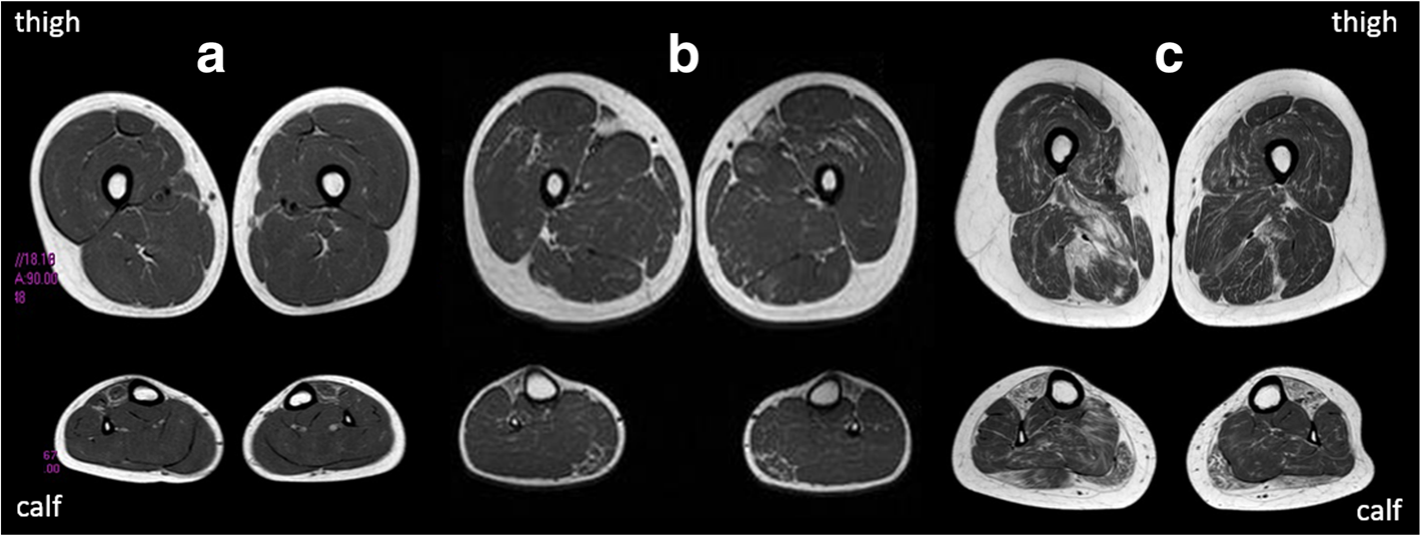
Muscle MRI of lower limb of three cases with different severity. Upper images refer to thigh, whereas lower images refer to calf muscles. Case 7 in (**a**) is the less affected and only initial fat substitution of tibialis anterior is noted. This feature is shared by all the patients. Interesting, in this case only, the tibialis involvement seems to start from the periphery of the muscle, a feature which has been already described in quadriceps muscles of COL6 mutated patients. In (**b**) involvement of medial gastrocnemius and very initial involvement of vastus medialis is present. This MRI belongs to case 6 at age 16. In (**c**) the tibialis anterior is totally replaced by fat tissue. Medial and lateral gastrocnemii are also affected. In the thight, vastus medialis and adductor magnus are involved but rectus femori is spared. This is case 5 and MRI was performed at 44 years of age

Pelvis, thigh and lower leg T_1_w images were reassessed in 13 of 20 patients in whom myoimaging had been performed (Fig. 4, Additional file 3: Table S3 and Additional file 4: Figure S9).

All the patients showed tibilias anterior as the most affected muscle followed by extensor digitorium longus, with relative sparing of the peroneal group. This was independent from age at muscle MRI. Gastrocnemii were involved in about half of the patients with a slight predominance of the medial gastrocnemius.

Muscle MRI of the thigh showed prevalent antero-medial involvement with vastus medialis, adductor magnus and sartorius affected in one third of patients; vastus intermedius, semimembranosus and biceps femoris were also affected, at a lesser extent. Rectus femoris was usually spared with the only exception of case 16, 17 and 21 where there was complete substitution of all the thigh muscles.

### Molecular analyses

**Fig. 5:**
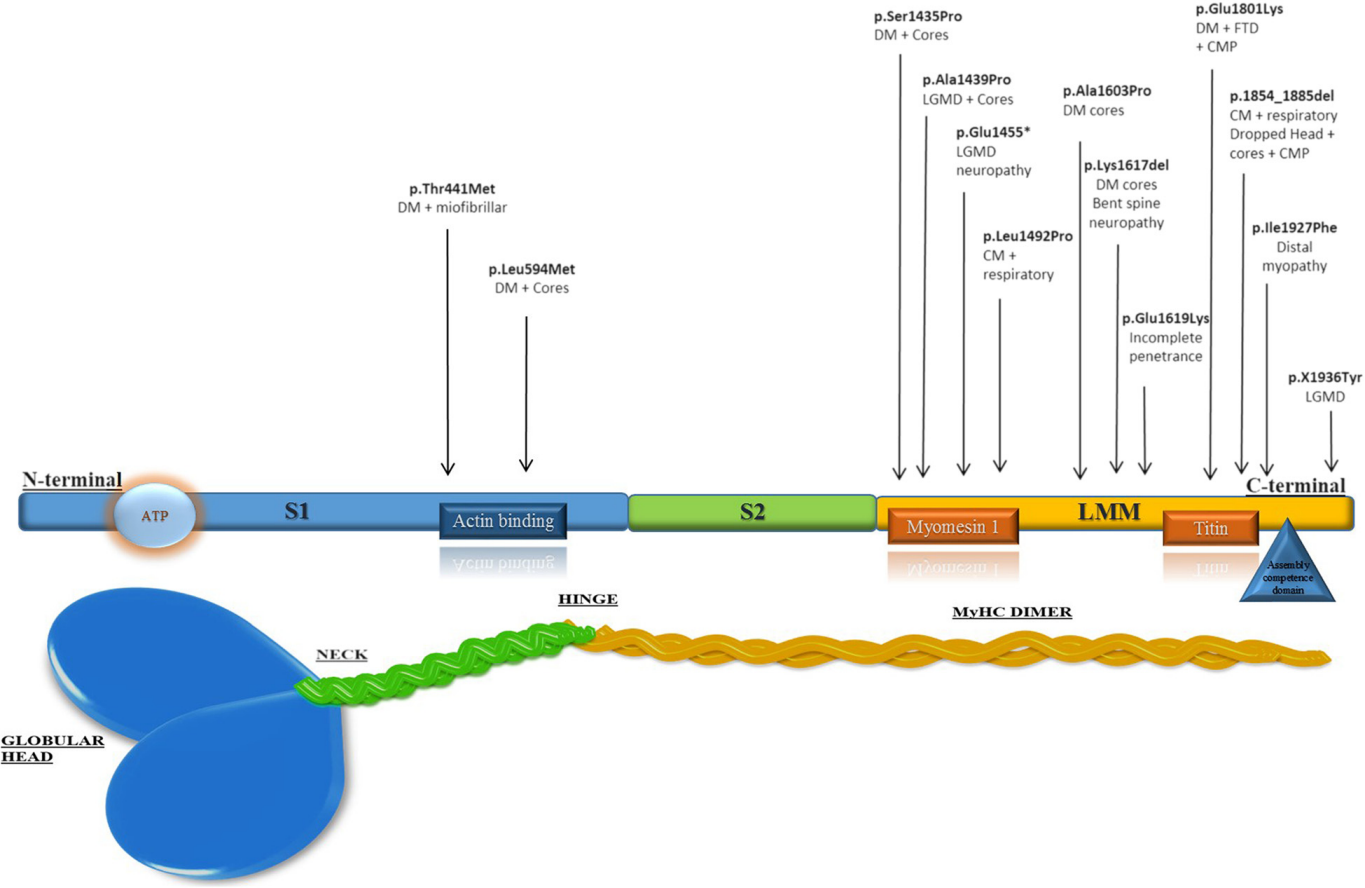
Morbidity map. Schematic representation of the MYH7 protein with its domain and distribution of the mutations described in the study. Most mutations are localized in the C-terminus of the protein. Near each mutation, phenotype is briefly described. DM distal myopathy; CMP cardiomyopathy; CM congenital myopathy; LGMD limb girdle muscular dystrophy; FTD fibre type disproportion

We identified 14 heterozygous mutations in *MYH7*. Most were missense affecting the C-terminal rod domain between exons 31-39 (LMM domain). Two missense mutations are located in exons 14 and 16 thus affecting the NH domains. Additional file 5: Table S4 summarizes the mutations identified in this study whereas Fig. 5 depicts how they are distributed along the presumed protein structure.

#### Novel mutations

Four novel mutations, including two missense, a change affecting the splice site junction and a variant affecting the stop codon (stop loss) were identified.

In particular, a novel change in exon 31 (c.4303 T > C, p.Ser1435Pro) was detected in Family 1. Patient 6 showed a novel c.5655 + 1G > A variant leading to an in-frame skipping of exon 38 (Additional file 6: Figure S7). A novel stoploss mutation (c.5808G > C/p.X1936Tyr) was identified in patient 14. The variant is predicted to determine the addition of 31 extra amino acids to the encoded protein (p.X1936YfsX32). Lastly, patient 16 showed an unreported missense variant (c.1780C > A p.Leu594Met). The variant, affecting an amino acid conserved during the evolution, is not reported in public databases collecting NGS data (ExAC, 1000Genomes or Exome Variant Server) and all the *in silico* tools used for its interpretation (SIFT, Polyphen, Mutation Taster) predicted a putative damaging effect. Even if a segregation analysis is hampered by the lack of further relatives, we considered the variant probably causative of the phenotype observed in patient 16.

#### Previously described mutations

We identified ten already described mutations. In the affected members of family 2 (case 5-6) and in patients 13 and 15, we identified already described changes to Proline [16, 17]. A different missense variant (c.5401G > A/p.Glu1801Lys) segregating with the disease was identified in members of Family 3, as reported [7].

Two missense mutations (c.5779A > T/p.Ile1927Phe and c.1322C > T/p.Thr441Met) were identified in patients 17 and 21. These variants have previously been associated with cardiomyopathy [18–20] a clinical feature not detected in our two patients. A similar scenario occurs for the nonsense c.4363G > T/p.Glu1455*, already reported elsewhere [21].

The recurrent p.Lys1617del [17] was found in three different patients (case 4, 8 and 19). A *de novo* synonymous mutation c.5655G > A/p.Ala1885Ala was identified in patient 18. The variant, very recently described by Pajusalu [22], causes an in-frame skipping of exon 38 (Additional file 6: Figure S7). Consequently, the encoded protein is predicted to be 32 amino acids shorter than the wild type. Both the c.5655G > A and the c.5655 + 1G > A mutations (identified in patients 18 and 7, respectively) cause an identical effect on the protein sequence (p.1854_1885del).

#### Functional analysis of mutations of the LMM domain

We performed functional prediction studies using the COILS program of the following mutations: p.Ser1435Pro, p.Ala1439Pro, p.Ala1603Pro and p.Glu1619Lys.

All mutations affect the ability of the protein to form stable and functional thick filament, changing the shape of the heptad repeat motif in the LMM region. (Additional file 7: Figure S6)

## Discussion

### MYH7 mutations often lead to a distal myopathy starting at lower limb

To date 327 mutations in *MYH7* have been reported in the related mutation database, myoMAPR (http://bmf2.colorado.edu/myomapr/) and it has becoming clear that alterations in *MYH7* are no longer an uncommon cause of skeletal myopathy. The forms of MSM and LDM are fronting a growing number of isolated or familial cases, which do not exhibit distal muscle weakness or do not have hyaline bodies nor other myofibrillar alteration in muscle biopsy [4, 5]. In their place, mini-cores and core myopathy or features of FTD have been commonly reported. Recently Lamont and colleagues described well this broadened spectrum of MYH7-related myopathies reporting findings in families with known and novel mutations in MYH7 [23]. Our data largely matches Lamont work in that the LDM form is definitely more common than MSM, being present here as the predominant phenotype in 19 of 21 patients. However since onset can be in adult and in childhood we preferred here to use the general term distal myopathy (DM). Similar to the Australian multicenter study, we found that axial involvement is common (42 %) and can seldom be the prime feature presenting with bent spine and dropped head as in cases 7 and 8. Also, we observed that the distal myopathy usually starts in the lower limbs leading to foot deformities.

### *MYH7* cardiomyopathy is common in male and can be also due to mutations in tail regions

Whilst clinical presentation of MYH7 skeletal myopathy is starting to be better clarified, more complexity arises with the concomitant presence of cardiomyopathy. It was initially postulated that mutations in the NH globular head mainly led to a cardiomyopathy phenotype, whereas mutations in the COOH tail domain, in particular the LMM domain, were responsible for the skeletal myopathies variably associated with cardiomyopathy. This distinction is no longer pertinent as several cases with skeletal myopathies and mutations in the globular head have been reported [24], usually presenting associated cardiac involvement [18, 25]. On the other hand, several reports of cardiomyopathy and mutations in the tail COOH domain of the protein have also been described [7, 9, 23]. In our cohort, cardiomyopathy occurred in seven cases and uniquely associated with tail domain mutations. Conversely, the only two patients who harbored mutations in the globular head did not manifest heart disease. Of note, clinically relevant cardiomyopathy is common in men from our study group and this does not seem to correlate with the age at disease manifestations. Factors associated with male hormone might be hypothesized to play a role, whereas genetic modifiers associated with X-chromosome are ruled out by the occurrence of a male-to-male transmission in fewer families (see also Fig. 1- Family 3). On the other hand, several mutations previously reported associated with cardiomyopathy are not causing cardiac manifestation in our patients. If these data point to the difficulties in phenotype-genotype correlations, little is usually reported on the neurological and neuromuscular examinations of patients described with a predominant heart involvement and we cannot exclude the presence of subtle features affecting skeletal muscles.

### *MYH7* cases have interfamilial variability and less intra-familial variability

Several previous reports describe familial variability in MYH7-related myopathy [3, 26, 27]. Conversely, in our study, we observed rather homogeneous phenotype, albeit this of course cannot be applied to later onset forms, where younger patients present only a part of the full clinical spectrum. There are two reports of recessive form of *MYH7* related myopathies [8, 9]. In both studies, patients carry homozygous mutations and display a scapulo-peroneal myosin storage phenotype associated with cardiomyopathy. We have not identified any recessive family; however, case 8 in our study carries two different heterozygous mutations leaving open the possibility of an AR inheritance.

In our cohort, the most severely affected patients (case 15 and 18) have early onset with diffuse muscle weakness and wasting, progressive scoliosis and respiratory involvement very much resembling congenital myopathies without cardiac involvement. At the opposite spectrum, three patients (case 1, 9, 13) have mainly distal weakness with onset in later adulthood similar to most common neuropathies. Two of these patients presented cores in muscle biopsy, whereas the third has features of FTD and severe cardiac involvement (case 9). A milder and mainly proximal weakness is also described in a 60-year-old patient (case 14) with unspecific changes at muscle biopsy.

### *MYH7* myopathy often shows progression of symptoms and later cardiac involvement

All patients presented in this study have a progressive course of the disease sometimes with proximal involvement in later stages as suggested also by Lamont [23]. Certainly a proximal weakness is common in older patients of our cohort, whereas congenital or childhood cases usually manifest a typical atrophy of the anterior lower leg compartment and hypertrophy of posterior leg. Moreover, cardiac manifestations usually affect adult population or develop after the skeletal myopathy. Case 12 represents the exception to this rule; he presented at the age of 7 years only with initial signs of LVNC cardiomyopathy and elevated CK, without overt muscle weakness. His father and grandfather (case 9 and 10) had classical LDM, a more severe cardiomyopathy and required pacemaker implantation. A similar report has been described in a five-generation family with a progressive cardiac involvement ranging from relaxation abnormality to noncompaction, ventricular arrhythmias, and dilated cardiomyopathy [28].

All these considerations propose a continuum in the disease spectrum related to *MYH7* mutations from early onset forms with features resembling a congenital myopathy to adult forms with also proximal involvement and heart disease.

Disease progression can be also depicted by evolution of the muscle histopathology as shown in case 4. In this patient clear cores and mini-cores lesions were evident in the first biopsy (Fig. 3 g-l and Additional file 2: Figure S8). Ten years later, a second biopsy from the contralateral muscle failed to identify cores and showed a diffuse alteration of the myofibrillar network. Modification of muscle damage in congenital myopathies overtime is not a new concept, being described already for cores and nemaline biopsies [29].

### Core pathology is common in muscle biopsies from *MYH7* mutated cases

Our revision of muscle biopsies from 16 patients showed that the LDM phenotype is often associated with core pathology, and FTD is another common histopathology feature. In general, histological analyses in our cohort suggest an overlap with the spectrum of congenital myopathies as already indicated by description of central cores in *MYH7*-related hypertrophic cardiomyopathy and by several familial reports with FTD [6, 7, 22]. On the contrary, only one of the muscle biopsies in our series met the pathological criteria for hyaline bodies/myosin storage and abnormal expression of slow myosin in myofilaments was not encountered. This result cannot be explained with differet genotypes, as most of the previous mutations associated with hyaline bodies were localized in the tail domain of slow myosin [30, 31], which is the most affected domain also in our study.

### Novel mutations and genotype-phenotype correlation in comparison to literature

Our study also added a second case with the c.5655G > A variation in exon 38 (case 18) after the case described by Pajusalu et al [22] and a further *de novo* splicing mutation (c.5655 + 1G > A) affecting intron 38 in case 7. In both cases, as expected, we confirmed an in-frame skipping of the exon 38 upon mRNA analyses in skeletal muscle Additional file 6: Figure S7). The subsequent lack of 32 amino acids in the myosin tail (p.1854_1885del) may probably hamper the correct assembly of the myosin filament as suggested [21]. Albeit they presented a similar pathobiological mechanism, the two patients, showed different phenotypes: a dropped head due to axial involvement together with heart dysfunction in case 7 and an early infantile onset of respiratory muscle impairment in case 18..[22]. Most important in the muscle biopsies of case 7 and 18 there were no features of FTD which characterized the Estonian boy identified by exome sequencing reported in [22].

In seven patients (of which three in family 1 and two in family 2) we identified different missense variants in the tail LMM domain changing highly conserved amino acids to proline and likely influencing protein domain structure, including the novel c.4303 T > C p.Ser1435Pro (Family 1). Previous works reported that mutations in the LMM region might affect the ability of the protein to form stable and functional thick filament, changing the shape of the heptad repeat motif.

### Complexity of *MYH7* differential diagnosis and the role of muscle MRI

Considering this broad and overlapping spectrum of clinical presentations, genetic analyses of MYH7-related phenotypes can be difficult to target and it is not surprising to have a delay in the conclusive diagnosis [28]. From our cohort, average time to diagnosis from onset of symptoms was nearly 20 years and 10 patients were identified only after of the study of numerous genes associated with congenital myopathies. The typical presentation of foot-drop in LDM can lead to an initial misdiagnosis of Hereditary Motor and Sensory Neuropathy, as happened in the original Australian family [32]. However compensatory calf hypertrophy and severe weakness of wrist and fingers extensor complete the phenotype. These features can help in the differential diagnosis with motor neuropathies (case 13) where both the anterior and posterior compartment of leg are involved as well as intrinsic muscles of hands before flexors.

Muscle MRI of lower limbs in previously reported patients harboring mutations in *MYH7* demonstrated a typical pattern consisting in early and more severe involvement of the tibialis anterior muscle, followed by extensor hallucis longus and extensor digitorum longus muscles, whereas the lateral gastrocnemius muscle was always spared [33, 34]. In the thighs, the vastus intermedius and lateralis were the most affected muscles; followed by the biceps femoris and semimembranosus muscles, whereas the rectus femoris, adductor longus, semitendinosus, and gracilis muscles were usually spared (Additional file 4: Figure S9) [3]. Slightly different from previous reports, we noticed quite an earlier involvement also of vastus medialis and sartorius, whereas vastus intermedius, semimebranosus and biceps femoris were affected later. Interestingly, a progression in the muscle MRI pattern can be drawn from an initial substitution of antero-medial muscles (Fig. 4), followed by the involvement of postero-lateral muscles.

More relevant MRI finding in the 13 patients from our study corroborated the notion that early involvement of the tibialis anterior muscle can be considered the “red flag” of a myosinopathy, also in patients with atypical features, with a pattern of involvement that reflects the clinical progression from distal to proximal muscle weakness.

However, considering the wide phenotypic spectrum related to *MYH7*-myopathyes, a distinction from other congenital myopathies with distal affection is essential. Particularly a predominant anterior lower leg involvement is always presents in *NEB*-myopathies and in *ACTA1*-myopathies with tibialis anterior involved first [35], but the different histological framework at muscle biopsy and the prevalent involvement at thigh level of posterior compartment in *ACTA1* and *NEB* mutations, comparing with the prevalent involvement of vasti muscles in myosinopathies could direct genetic tests. On the other and, in cases of *MYH-7* mutations and predominant proximal or axial weakness the striking affection of tibialis anterior compared to thigh involvement is strongly suggestive. Finally, few patients in our cohort were first diagnosed as neuronopathies; also in these cases muscle MRI can play an important role in differential diagnosis considering the striking differences of the pattern of muscles involvement. In patients with distal spinal muscular atrophy (or SMALED), in fact, there is the prevalent involvement of thigh muscles with sparing or hypertrophy of adductor longus and tibialis anterior (i.e. *DYNC1H1* mutated patients) or of biceps femorii and medial gastrocnemius (i.e. *TRPV4* mutated patients) [36] differently from those described in *MYH-7* related myopathies.

## Conclusion

To sum up, this study describes a cohort of 21 Italian patients carrying a total of 14 different mutations in *MYH7* and displaying a broad spectrum of clinical presentations, such as different disease onset, muscle weakness distribution, and the co-occurrence of heart involvement. Under this blurred clinical-genetic scenario, we foresee three forms of MYH7-myopathies: 1) an early onset distal myopathy with cores, 2) a late onset form of distal myopathy without histological evidence of cores and with variable association of cardiomyopathy and/or FTD and 3) the least frequent form of limb girdle involvement with myofibrillar damage resembling the myosin storage myopathy. We confirm that sites of mutations do not correlate with severity and cannot predict phenotype. This is particularly true for cardiomyopathies which appear to be most common in men irrespective of genotype and unrelated to mutations falling in specific domains. Functional studies and more extensive molecular analyses are warrant to explore these aspects.

Unlikely the other, more frequent forms of congenital myopathy, our findings also propose a progressive nature of *MYH7*-related myopathies, and a striking difficulty in targeting the underlying molecular etiology. Thus, NGS tools such as MotorPlex can be dually useful to pinpoint the *MYH7* mutations in unspecific cases and to identify associated alterations in additional muscular genes that could act as modifiers and provide an explanation for different phenotype.

## Abbreviations

CMP, cardiomyopathy; DM, distal myopathy; FTD, fibre type disproportion; LDM, laing distal myopathy; LMM, light meromyosin domain; LVNC, left ventricule non compacted; MRI, magnetic resonance; MSM, myosin storage myopathy; NGS, next generation sequencing

## Additional files

Additional file 1: Table S2. Histopathological findings. FTD fibre type disproportion. (DOCX 20 kb) Additional file 2: Figure S8. Details of muscle biopsy from case 4, taken at 7 years old. A NADH staining (40x) from a longitudinal section showing the extent of minicores along the fibres in the subsarcolemmal area and diffuse in the sarcoplasm. B Electron microscopy images (7000x) from the same biopsy, showed few normal sarcomeres with regular alignment near a sharply demarcated core areas with myofibrillar disorganization and sarcomeres disruption. (TIF 1388 kb) Additional file 3: Table S3. Muscle MRI findings. AL: adductor longus; AM: adductor magnus; G: gracilis; GM: gastrocnemius medialis; PG: peroneal group; RF: rectus femoris; S: Sartorius; SO: soleus; TA: tibialis anterior; VL: vastus lateralis; VM: vastus medialis; VI: vastus intermedius; BF: biceps femoris; SM: semimembranousus. lc/bc: capus longus and brevis of biceps femori Mod: moderate, Net: no sign of infiltration/fat substitution (DOCX 23 kb) Additional file 4: Figure S9. Schematic representation of muscle involvement in MYH7 patients. In dark grey are the most affected muscles, in light grey the muscle which are still affected but to a lesser extent. In white are the spared muscles. AL: adductor longus; AM: adductor magnus; G: gracilis; GM: gastrocnemius medialis; PG: peroneal group; RF: rectus femoris; S: Sartorius; SO: soleus; TA: tibialis anterior; VL: vastus lateralis; VM: vastus medialis; VI: vastus intermedius; BF: biceps femoris; SM: semimembranousus. (TIF 270 kb) Additional file 5: Table S4. Type and site of mutations and previous literature reports. LMM ligh meromyosin domain. DM distal myopathy. LDM Laing distal myopathy. CM congenital myopathy. CMP cardiomyopathy. LVNC left ventricule non compacted. FTD fibre type disproportion [37]. (DOCX 22 kb) Additional file 6: Figure S7. Results of mRNA analysis on patients 7 and 18. PCR reactions, performed using cDNA synthesized from muscle total RNA of patients 7 (c.5655 + 1G > A) and 18 (c.5655G > A), result into an additional short fragment, because of an abnormal splicing. The sequences of the normal size fragment showed the normal exon37-exon38 boundary; electropherograms of the short fragment show the skipping of exon38 and the exon37-exon39 junction. Red circles indicate the genomic position of the mutated nucleotides. (JPG 514 kb) Additional file 7: Figure S6. COILS prediction of the effect of mutations in the LMM region on the probability of the myosin tails forming a coiled coil. COILS analysis of residues 1193-1805, using a MTIDK matrix and scanning windows of 14 (green line), 21 (blu line) and 28 (red line) amino acids, shows that all the 4 novel distal-myopathy mutations (p.Ser1435Pro, p.Ala1439Pro, p.Ala1603Pro and Glu1619Lys) impact the ability of the myosin tail to form a coiled coil (or hamper a coiled-coil conformation). Arrow indicates the position of the mutated amino acids. (JPG 183 kb)
