# Supplementary material for: MYH7-related myopathies: clinical, histopathological and imaging findings in a cohort of Italian patients
Source: Orphanet J Rare Dis. 2016 Jul 7;11:91. doi: 10.1186/s13023-016-0476-1 (PMC4936326; doi:10.1186/s13023-016-0476-1)
Supplement: Additional file 1: Table S2. — Histopathological findings. FTD fibre type disproportion. (DOCX 20 kb) [file 13023_2016_476_MOESM1_ESM.docx]

|  | Age at biopsy (yrs) | Muscle | Central nuclei | Cores in type I fibers | Cores in type II fibers | Type I fibers | Hyalin bodies | Increase of connective tissue | Myosin staining | Other features |
| --- | --- | --- | --- | --- | --- | --- | --- | --- | --- | --- |
| 1 |  | quadriceps | yes | yes | no | small |  | no |  |  |
| 2 |  | tibialis | yes | yes | no | small |  | yes |  |  |
| 4 | 7 and 15 | quadriceps | yes | yes (minicores) | yes (minicores) | FTD |  | moderate | normal |  |
| 5 | 45 | deltoid | rare | yes (minicores) | yes (minicores) | predominance |  | yes | normal | rimmed vacuoles |
| 7 | 5 | quadriceps | yes | yes (minicores) | yes (minicores) | predominance |  | mild | normal |  |
| 8 | 5 | biceps suralis | yes | yes | no | predominance |  | moderate | normal | angulated fibres |
| 9 |  | biceps brachii | yes | no | no | FTD |  | mild |  |  |
| 10 |  | biceps brachii | yes | no | no | FTD |  | no |  |  |
| 13 | 53 | biceps brach. | no | yes |  | small |  | No |  |  |
| 14 | 50 | quadriceps | no | no | no | hypertrophic |  | yes |  |  |
| 15 | 13 | biceps brachii |  |  |  |  |  |  |  | unspecific muscle biopsy |
| 16 | 37 | quadriceps |  | yes |  | predominance |  | yes |  |  |
| 17 | 9 |  | no | no | no |  |  | no |  | increase of lipid content |
| 18 | 10 | quadriceps | yes | no |  | no |  | no |  | type 2 predominance |
| 19 | 12 | quadriceps | no | yes (minicores) | no | no |  | no |  |  |
| 21 | 38 | biceps brachii | yes | no | no | no | yes | yes |  | angulated fibres |

**Table 2**

**Histopatological findings**

FTD: fibre type disproportion.
