# Supplementary figures and images for: MYH7-related myopathies: clinical, histopathological and imaging findings in a cohort of Italian patients
Source: Orphanet J Rare Dis. 2016 Jul 7;11:91. doi: 10.1186/s13023-016-0476-1 (PMC4936326; doi:10.1186/s13023-016-0476-1)

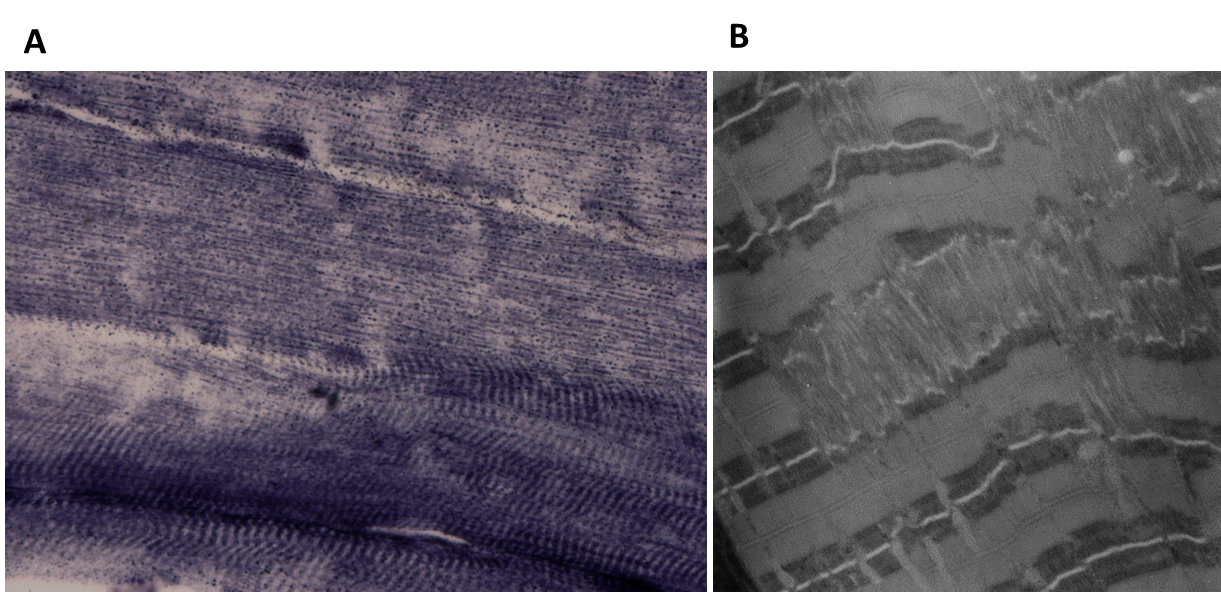

Supplement: Additional file 2: Figure S8. — Details of muscle biopsy from case 4, taken at 7 years old. A NADH staining (40x) from a longitudinal section showing the extent of minicores along the fibres in the subsarcolemmal area and diffuse in the sarcoplasm. B Electron microscopy images (7000x) from the same biopsy, showed few normal sarcomeres with regular alignment near a sharply demarcated core areas with myofibrillar disorganization and sarcomeres disruption. (TIF 1388 kb) [file 13023_2016_476_MOESM2_ESM.tif]

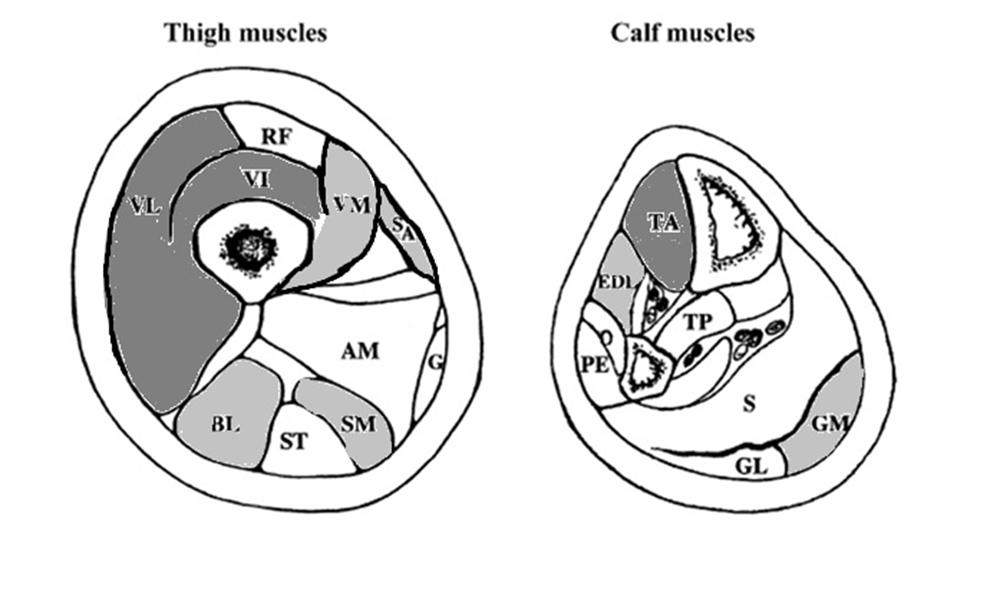

Supplement: Additional file 4: Figure S9. — Schematic representation of muscle involvement in MYH7 patients. In dark grey are the most affected muscles, in light grey the muscle which are still affected but to a lesser extent. In white are the spared muscles. AL: adductor longus; AM: adductor magnus; G: gracilis; GM: gastrocnemius medialis; PG: peroneal group; RF: rectus femoris; S: Sartorius; SO: soleus; TA: tibialis anterior; VL: vastus lateralis; VM: vastus medialis; VI: vastus intermedius; BF: biceps femoris; SM: semimembranousus. (TIF 270 kb) [file 13023_2016_476_MOESM4_ESM.tif]

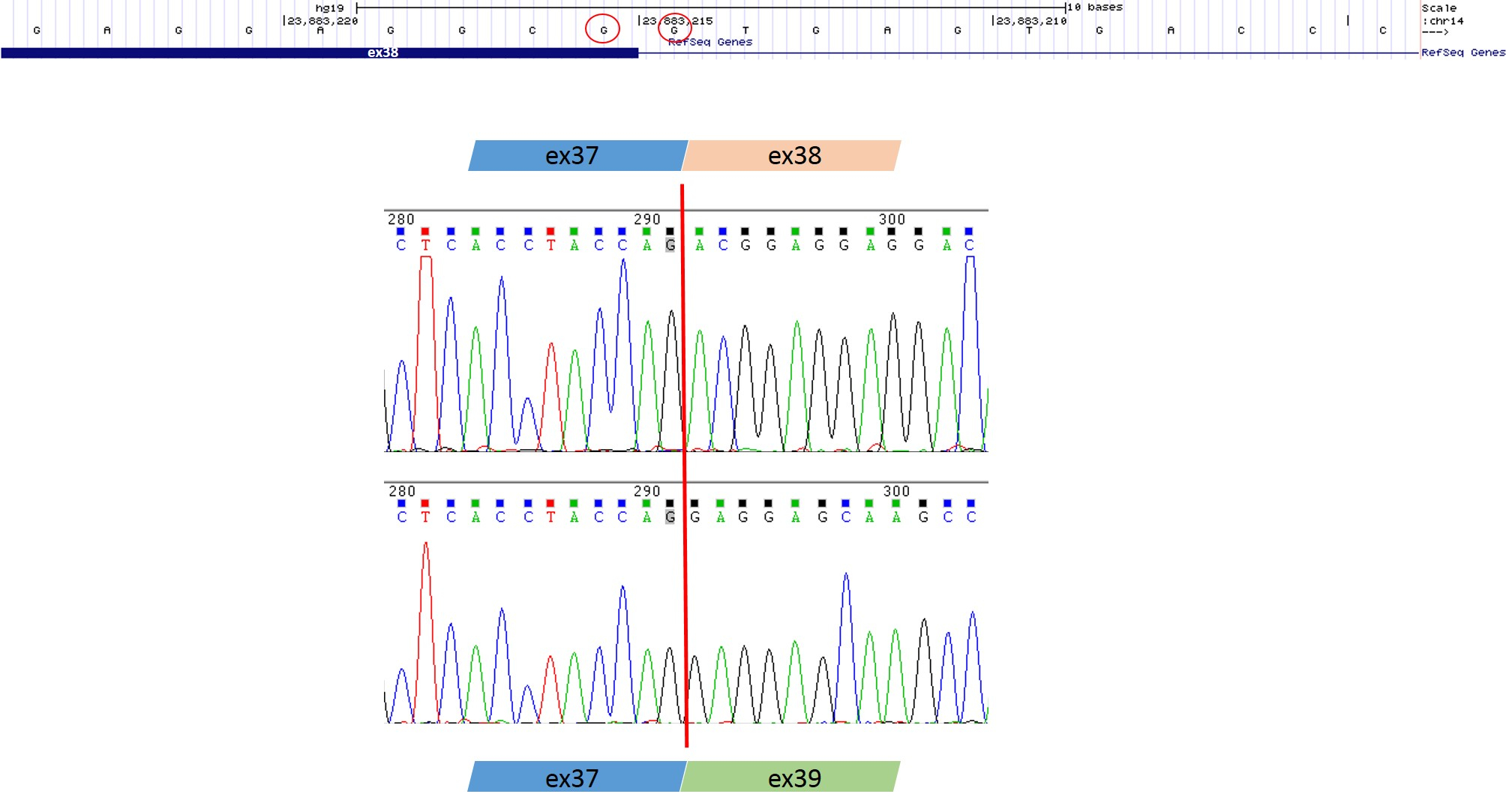

Supplement: Additional file 6: Figure S7. — Results of mRNA analysis on patients 7 and 18. PCR reactions, performed using cDNA synthesized from muscle total RNA of patients 7 (c.5655 + 1G > A) and 18 (c.5655G > A), result into an additional short fragment, because of an abnormal splicing. The sequences of the normal size fragment showed the normal exon37-exon38 boundary; electropherograms of the short fragment show the skipping of exon38 and the exon37-exon39 junction. Red circles indicate the genomic position of the mutated nucleotides. (JPG 514 kb) [file 13023_2016_476_MOESM6_ESM.jpg]

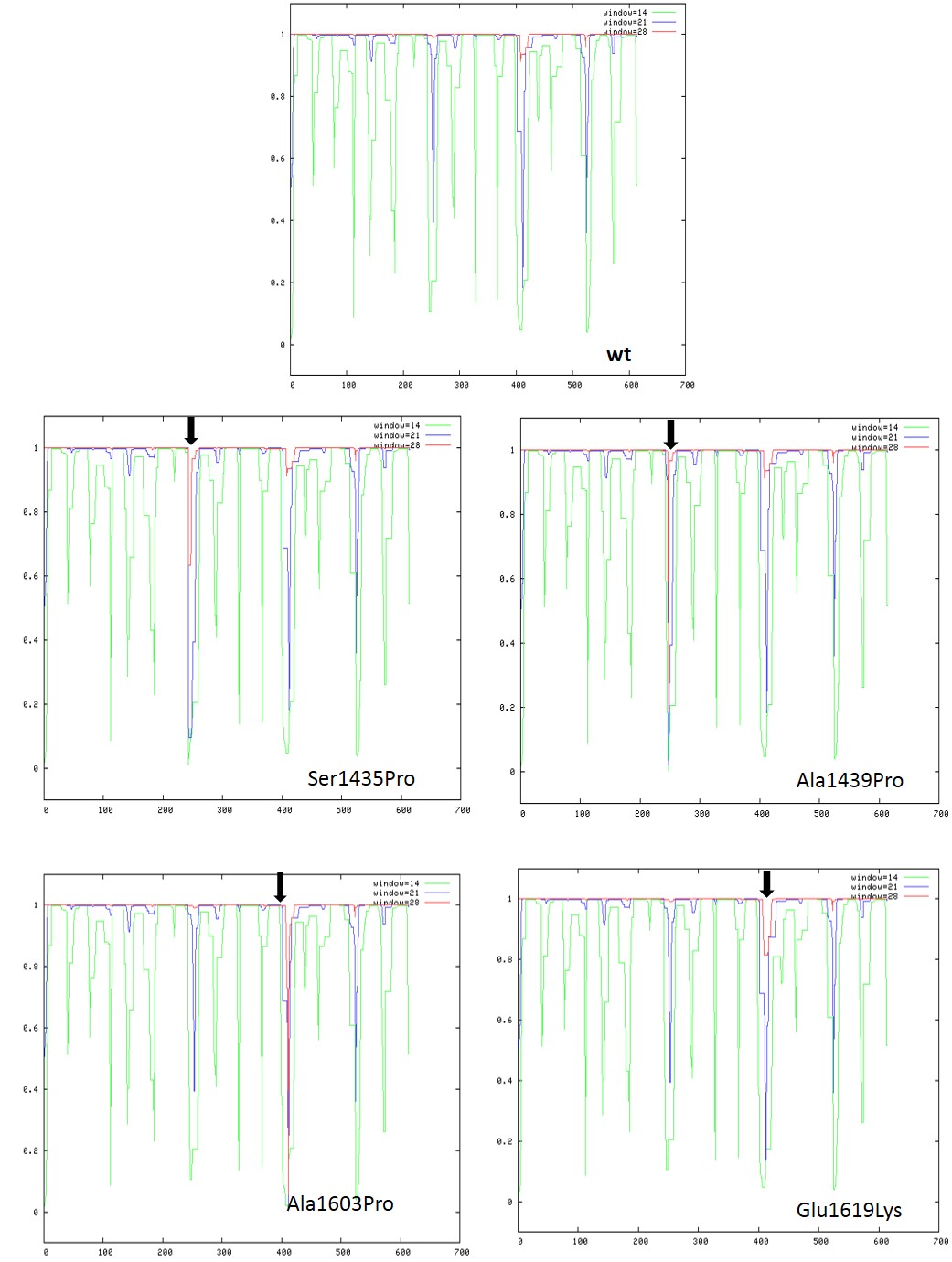

Supplement: Additional file 7: Figure S6. — COILS prediction of the effect of mutations in the LMM region on the probability of the myosin tails forming a coiled coil. COILS analysis of residues 1193-1805, using a MTIDK matrix and scanning windows of 14 (green line), 21 (blu line) and 28 (red line) amino acids, shows that all the 4 novel distal-myopathy mutations (p.Ser1435Pro, p.Ala1439Pro, p.Ala1603Pro and Glu1619Lys) impact the ability of the myosin tail to form a coiled coil (or hamper a coiled-coil conformation). Arrow indicates the position of the mutated amino acids. (JPG 183 kb) [file 13023_2016_476_MOESM7_ESM.jpg]
