# Supplementary material for: MYH7-related myopathies: clinical, histopathological and imaging findings in a cohort of Italian patients
Source: Orphanet J Rare Dis. 2016 Jul 7;11:91. doi: 10.1186/s13023-016-0476-1 (PMC4936326; doi:10.1186/s13023-016-0476-1)
Supplement: Additional file 3: Table S3. — Muscle MRI findings. AL: adductor longus; AM: adductor magnus; G: gracilis; GM: gastrocnemius medialis; PG: peroneal group; RF: rectus femoris; S: Sartorius; SO: soleus; TA: tibialis anterior; VL: vastus lateralis; VM: vastus medialis; VI: vastus intermedius; BF: biceps femoris; SM: semimembranousus. lc/bc: capus longus and brevis of biceps femori Mod: moderate, Net: no sign of infiltration/fat substitution (DOCX 23 kb) [file 13023_2016_476_MOESM3_ESM.docx]

| **Muscular involvement (T1 seq.)** | | | | | | | | | | | | | | | | | | | |
| --- | --- | --- | --- | --- | --- | --- | --- | --- | --- | --- | --- | --- | --- | --- | --- | --- | --- | --- | --- |
|  | Age at MRI | **RF** | **VL** | **VM** | **VI** | **AM** | **AL** | **S** | **G** | **BF** | **SM** | **ST** | **TA** | **EDL** | **PG** | **TP** | **SO** | **GM** | **GL** |
| **1** | 68 | net | net | mild | mild | mild | net | net | net | net | mild | mild | severe | mod | net | net | net | mild | net |
| **2** | 61 | net | net | net | net | net | net | net | net | net | net | net | severe | net | net | net | net | net | net |
| **3** | 35 | net | net | net | net | net | net | net | net | net | net | net | mod | mild | net | net | net | net | net |
| **4** | 15 | net | mild | mild | - | - | - | severe | net | - | - | - | severe | - | net | - | - | mod | net |
| **5** | 44 | net | mild | mild | mild | mild  (sx) mod  (dx) | net | severe(dx) mod  (sx) | mild | severe  (lc)  mild  (bc) | mod  (dx)  mild  (sx) | mild | severe | severe | net | net | mild | severe | mod |
| **6** | 14 | net | net | net | net | mild | net | mild | net | net | net | net | mild | mod | net | net | net | net | net |
| **7** | 6 | net | net | net | net | net | net | net | net | net | net | net | mod | mild | net | net | net | mild | mild |
| **12** | 53 | mild | severe | severe | severe | severe | severe | mild | mild | severe | severe | severe | severe | severe | net | net | net | severe | net |
| **15** | 16 | - | - | - | - | - | - | - | - | - | - | - | mod | - | - | - | mild | - | - |
| **16** | 37 | mod | mild |  |  |  |  |  |  | mild | mild | mild | severe | severe | severe | severe | mod | severe | severe |
| **17** | 35 | severe | mild | severe | severe | severe | severe | mild | mild | mod | severe | severe | severe |  | mod | severe | severe | severe | severe |
| **18** | 15 | net | net | net | net | net | net | net | net | net | net | net | severe | net | net | net | net | net | net |
| **19** | 24 | - | - | - | - | - | - | mild | - | - | - | - | mild | - | - | - | - | - | - |
| **21** | 38 | severe | severe | severe | severe | mod | mod | mild | mild | n | n | n | severe | severe | severe | mod | mod | mod | mod |

**Table 3**

**Muscle MRI findings**

AL: adductor longus; AM: adductor magnus; G: gracilis; GM: gastrocnemius medialis; PG: peroneal group; RF: rectus femoris; S: Sartorius; SO: soleus; TA: tibialis anterior; VL: vastus lateralis; VM: vastus medialis; VI: vastus intermedius; BF: biceps femoris; SM: semimembranousus. lc/bc: capus longus and brevis of biceps femori

Mod: moderate

Net: no sign of infiltration/fat substitution
