# Supplementary material for: MYH7-related myopathies: clinical, histopathological and imaging findings in a cohort of Italian patients
Source: Orphanet J Rare Dis. 2016 Jul 7;11:91. doi: 10.1186/s13023-016-0476-1 (PMC4936326; doi:10.1186/s13023-016-0476-1)
Supplement: Additional file 5: Table S4. — Type and site of mutations and previous literature reports. LMM ligh meromyosin domain. DM distal myopathy. LDM Laing distal myopathy. CM congenital myopathy. CMP cardiomyopathy. LVNC left ventricule non compacted. FTD fibre type disproportion [37]. (DOCX 22 kb) [file 13023_2016_476_MOESM5_ESM.docx]

|  |  | **mutation** | **protein** |  | **exon** | **domain** | **references** | **phenotype** |
| --- | --- | --- | --- | --- | --- | --- | --- | --- |
| 1 | chr14:23886762 | c.4303T>C | p.Ser1435Pro | missense | 31 | LMM | **this work** | DM + Cores (late onset) |
| 2 | chr14:23885313 | c.4303T>C | p.Ser1435Pro | missense | 31 | LMM | **this work** | DM + Cores (late onset) |
| 3 | chr14:23885313 | c.4303T>C | p.Ser1435Pro | missense | 31 | LMM | **this work** | DM + Cores (late onset) |
| 4 | chr14:23885313 | c.4850_4852del | p.Lys1617del | in-frame deletion | 34 | LMM | Meredith 2004 [36] (LDM) | DM + Cores |
| 5 | chr14:23885359 | c.4807G>C | p.Ala1603Pro | missense | 34 | LMM | Udd 2009 [11] (LDM) | DM + Cores |
| 6 | chr14:23885359 | c.4807G>C | p.Ala1603Pro | missense | 34 | LMM | Udd 2009 [11] (LDM) | DM + Cores |
| 7 | chr14:23883215 | c.5655+1G>A | p.1854_1885del | in-frame misplicing | int38 | LMM | **this work** | Dropped head + Cores +CMP |
| 8 | chr14:23885313 | c.4850_4852del | p.Lys1617del | in-frame deletion | 34 | LMM | Meredith 2004 [36] (LDM) | DM+neuropathy+bent spine |
| 8 | chr14:23885311 | c.4855G>A | p.Glu1619Lys | missense | 34 | LMM | Hershberger 2008 [37] (CMP) | DM+neuropathy+bent spine |
| 9 | chr14:23884362 | c.5401G>A | p.Glu1801Lys | missense | 37 | LMM | Ruggiero 2014 [7] | DM+FTD+CMP |
| 10 | chr14:23884362 | c.5401G>A | p.Glu1801Lys | missense | 37 | LMM | Ruggiero 2014 [7] | DM+FTD+CMP |
| 11 | chr14:23884362 | c.5401G>A | p.Glu1801Lys | missense | 37 | LMM | Ruggiero 2014 [7] | DM+CMP |
| 12 | chr14:23884363 | c.5401G>A | p.Glu1801Lys | missense | 37 | LMM | Ruggiero 2014 [7] | CMP only |
| 13 | chr14:23886750 | c.4315G>C | p.Ala1439Pro | missense | 31 | LMM | Park 2013 [16] | LGMD + Cores (late onset) |
| 14 | chr14:23886518 | c.4363G>T | p.Glu1455X | nonsense | 32 | LMM | Girolami 2010 [21](CMP) | LGMD + neuropathy |
| 15 | chr14:23886406 | c.4475T>C | p.Leu1492Pro | missense | 32 | LMM | Roncarati, R. 2011 [17] (CMP) | CM+ respiratory |
| 16 | chr14:23896902 | c.1780C>A | p.Leu594Met | missense | 16 | Head | **this work** | DM+ Cores |
| 17 | chr14:23882979 | c.5779A>T | p.Ile1927Phe | missense | 39 | LMM | Fokstuen 2008 [20] (CMP) | DM |
| 18 | chr14:23883216 | c.5655G>A | p.1854_1885del | in-frame misplicing | 38 | LMM | Pajusalu 2015 [22] (DM + FTD) | CM + respiratory |
| 19 | chr14:23885313 | c.4850_4852del | p.Lys1617del | in-frame deletion | 34 | LMM | Lamont 2014 [23] (LDM,CMP) | DM+ Cores |
| 20 | chr14:23882063 | c.5808G>C | p.X1936Tyr | stoploss | 40 | LMM | **this work** | LGMD |
| 21 |  | c.1322C>T | p.Thr441Met | missense | 14 | Head | Darin 2007 [19] (MPD + CMP) | DM + myofibrillar |

**Table 4**

Type and site of mutations and previous literature reports. LMM ligh meromyosin domain, DM distal myopathy, LDM: Laing distal myopathy. CM congenital myopathy, CMP cardiomyopathy, LVNC left ventricule non compacted. FTD fibre type disproportion
